# Recent progress in on-surface synthesis of nanoporous graphene materials

**DOI:** 10.1038/s42004-024-01222-2

**Published:** 2024-07-08

**Authors:** Tianchen Qin, Tao Wang, Junfa Zhu

**Affiliations:** 1 Department of Pharmacy, The First Affiliated Hospital of USTC, Division of Life Sciences and Medicine, and National Synchrotron Radiation Laboratory, University of Science and Technology of China, Hefei, 230029 P. R. China; 2 State Key Laboratory of Organometallic Chemistry, Shanghai Institute of Organic Chemistry, Chinese Academy of Sciences, Shanghai, 200032 P. R. China

**Keywords:** Reaction kinetics and dynamics, Synthesis of graphene, Scanning probe microscopy

## Abstract

Nanoporous graphene (NPG) materials are generated by removing internal degree-3 vertices from graphene and introducing nanopores with specific topological structures, which have been widely explored and exploited for applications in electronic devices, membranes, and energy storage. The inherent properties of NPGs, such as the band structures, field effect mobilities and topological properties, are crucially determined by the geometric structure of nanopores. On-surface synthesis is an emerging strategy to fabricate low-dimensional carbon nanostructures with atomic precision. In this review, we introduce the progress of on-surface synthesis of atomically precise NPGs, and classify NPGs from the aspects of element types, topological structures, pore shapes, and synthesis strategies. We aim to provide a comprehensive overview of the recent advancements, promoting interdisciplinary collaboration to further advance the synthesis and applications of NPGs.

## Abstract (web summary)

On-surface synthesis is a useful approach for the construction of nanoporous graphene materials, which are in turn of interest for various electronic applications. Here, the authors review the latest developments in the on-surface synthesis of atomically precise pristine and hetero-atom doped nanoporous graphene materials.

## Introduction

Graphene, a well-known two-dimensional carbon-based material, exhibits exciting electronic, optical, mechanical, and thermal properties owing to its unique structure, i.e., one-atom thickness, global sp^2^-hybridization, and honey-comb lattices^1–4^. The fully sp^2^-hybridized carbon atoms result in all of the C–C bonds being equivalent and alternated. Thus graphene is a zero-gap semimetal with a tiny overlap between valence and conduction bands^5^. However, the zero-gap character of graphene strongly limited its applications as a semiconductor material, as typically used in photodetectors, sensors, and particularly field effect transistors (FETs)^6,7^. Promising strategies to open the band gap and tune the electronic properties of graphene include dimension reduction, chemical functionalization, isotopes or heteroatom substitution, and controllable defects insertion^8–12^. Among these strategies, nanoporous graphene (NPG) containing periodic nanopores with specific topologies has emerged as a new candidate for tuning the bandgap and electronic structure of graphene, which typically have intriguing electronic and magnetic properties as determined by the structural topologies and the porous shapes^13^.

The geometric properties of two-dimensional (2D) carbon-based nanostructures play a critical role in determining their chemical and physical properties^8,13–16^. Specifically, electronic properties (such as band gap, the electronic states of frontier orbitals, and topological properties) of NPG can be tuned by rationally designing the size, shape, periodicity of nanopores, and specific edge geometry, as well as the degree of conjugation of building blocks^1,12,17–20^. The bandgap of NPG is particularly influenced by the localization of π-electron, which can be tuned by the controllable introduction of nanopores, for example, the larger delocalization of π-electrons induces the smaller bandgap^6,21,22^. Recently, as to how the topology affects the bandgap of NPG, several studies have tried to uncover the relationships between geometry, periodicity, and C–C bond density between building blocks from different perspectives^18–20,23,24^.

NPGs have traditionally been fabricated using techniques such as nanoimprint lithography^21^, combustion^25^, chemical vapor deposition^26^, and in-solution methods^27,28^. These methods have allowed for the widespread exploration of NPGs in various applications, particularly in molecular/ionic sieving and energy storage. Efficient molecular/ionic sieving requires two key factors: high selectivity and permeability. NPG is well-suited for this purpose due to its ability to balance these factors through the use of tunable nanopores and its single-atom thickness^29–42^. Boutilier et al. fabricated and evaluated the centimeter-scale nanoporous graphene membranes with subnanometer pores for gas separation applications^43^. They found that their gas separation performance for H_2_/CH_4_ nearly reached the Robeson limit, and H_2_/CO_2_ exceeded the Robeson limit. NPG, with specific architectures, also offers numerous advantages in energy storage. It possesses an ultrahigh surface area and excellent electrical conductivity^44–47^. By acting as electrode materials in supercapacitors and sodium-ion batteries, porous graphene enables high-rate capability and demonstrates excellent cycling performance^48,49^. Different from the above applications, the applications in electronics and quantum devices typically require single-layer NPG with atomic precision, because a subtle modification of structure may give rise to a significant change of the electronic and spin properties^50–52^. Nanoimprint lithography, combustion, and chemical vapor deposition lack atomic precision, while in-solution methods have limitations of solubility for the synthesis of two-dimensional materials. Therefore, the development of a synthesis strategy with atomic precision is highly anticipated.

Starting from well-designed small precursors, the bottom-up on-surface synthesis strategies provide an unprecedented route for synthesizing complex and atomically precise low-dimensional carbon-based nanostructures^53–64^. Using these strategies, great success in the synthesis of zero-dimensional nanographene and one-dimensional graphene nanoribbons (GNRs) have already achieved^10,65–72^. Although there still exists enormous challenges, in recent years, an increasing number of high-quality NPGs with various geometric structures have been fabricated^73,74^. This is mainly benefited from the rapid development of on-surface synthesis and in-depth understanding of reaction mechanism and controlling strategies, The atomically precise and tunable nanopores result in the NPGs serving as a platform for electronic applications which require certain band structures and electronic transportation properties^75–78^. Especially, the tunable bandgaps, semiconducting properties, and intrinsic electron–hole symmetry make NPG eminently suitable for applications in FETs^6,79–81^. Due to the local confinement of π-electrons induced by nanopores and the in-plane anisotropy of partial NPGs, GNRs with particular edge topology as the building block in these NPGs have been theoretically and experimentally demonstrated to act as one-dimensional (1D) electronic nanochannels that allow the preferential movement of electrons^76,82,83^. Moreover, the specific geometry, edge structure, and periodicity of regular pores significantly impact the topological states of NPG, among which honeycomb and Kagome lattice are the most representative examples^84–86^.

**Fig. 1:**
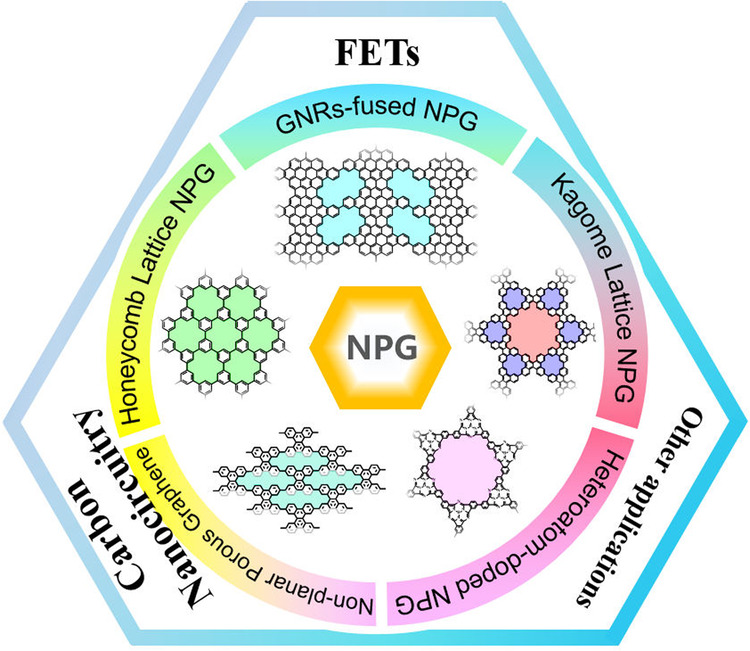
The scope of this review. The inner circle represents some typical classifications of NPGs and some representative works, while the outer circle showcases some applications of NPGs in the field of electronic devices. Representative classification of NPGs: honeycomb lattice NPG, Kagome lattice NPG, non-planar porous graphene, GNRs-fused NPG, and heteroatom-doped NPG. Typical applications in the field of electronic devices: FETs, carbon nanocircuitry and others.

Because of the enormous promising applications of NPGs, the synthesis of NPGs with atomic precision and high quality has evolved into one of the most active and challenging topics. In this review, we summarize the latest developments in the theoretical and experimental studies of NPGs. The scope of this review is summarized in Fig. 1. We first start with the types of elements, dividing NPG into two categories: pristine and heteroatom-doped NPGs. Secondly, in order to emphasize the structure–property relationship, we categorize the pristine NPGs into several types. Two basic points that affect the properties of NPG are their structural topology/geometry and the shape of the pores. Therefore, currently reported pristine NPGs can be further categorized into honeycomb lattice and Kagome lattice NPGs based on their geometry. In addition, in terms of the shape of the pores, we divide NPG into two categories: NPGs containing planar and nonplanar pores. Lastly, we emphasize the NPG fused by GNRs as a special NPG type, which have up to date the highest quality among NPGs and obtained by a unique hierarchical synthetic approach via on-surface synthesis. Building upon these successful synthetic strategies, we then discuss the recent progress of the applications of NPG, with a particular focus on FETs and other areas related to electronic devices. Finally, we provide a perspective of the present limitations and future developing directions of NPG.

## Recent progress in the on-surface synthesis of NPG

### Pristine NPGs

**Fig. 2:**
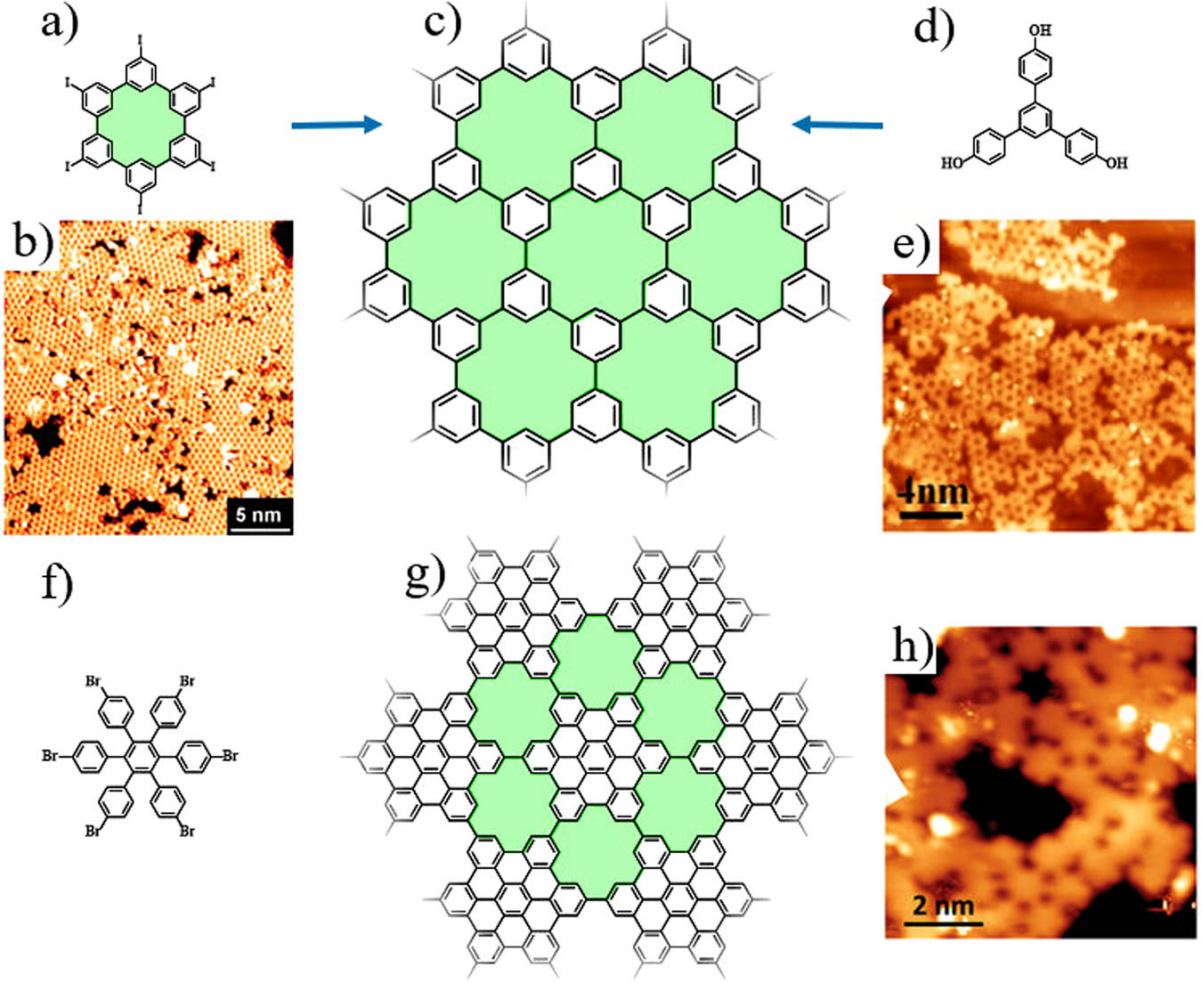
Structural models and STM images of some typical pristine NPGs. **a**–**e** On-surface synthesis of [18]annulene porous NPG whose molecular structure shown in (**c**): **b** and **e** are the large-scale STM images of [18]annulene porous NPG on Ag(111) obtained via the Ullmann coupling reaction from the cyclohexa-m-phenylene precursor (**a**), and via selective C–H bond activation from the 4,4‴-dihydroxy-p-quaterphenyl precursor (**d**), respectively. **b** Adapted with permission from ref. ^89^ ©2009 The Royal Society of Chemistry; **e** adapted with permission from ref. ^88^. ©2016 American Chemical Society). **f**–**h** On-surface synthesis of [18]annulene porous NPG whose molecular structure shown in **g** via hierarchical C–C coupling and cyclodehydrogenation by using hexakis(4-bromophenyl)benzene precursor **f** on Au(111), as revealed by the STM image in (**h**). (adapted with permission from ref. ^24^. ©2021 John Wiley & Son).

One of the pioneer’s works in on-surface synthesis of NPG with atomic precision was found by using hexaiodo-substituted macrocycle cyclohexa-m-phenylene as the precursor (Fig. 2a), 2009 Bieri et al. successfully constructed the super-honeycomb NPG via Ullmann coupling on an Ag(111) surface (Fig. 2b, c)^87^. Since then, a series of NPGs with various structures and degrees of conjugation have been gradually synthesized on surfaces^24,88,89^. Based on a series of three-halogenated phenylbenzene precursors, the honeycombed NPGs have been conducted via Ullmann coupling^90,91^. The NPGs containing [18]annulene pores have attracted most investigations due to their stable framework as well as the simple and feasible synthesis strategies. [18]Annulene pores can be regarded as the removal of six internal degree-3 vertices from the graphene skeleton. Li et al. synthesized [18]annulene pores doped NPG relying on the *ortho* C–H activations on Ag(111) (Fig. 2d, e)^88^. Our group synthesized the NPG with high inter-molecule bond density originating from a planar halogen-substituted nanographene molecular precursor while studying how to regulate the reaction pathway by using the basic kinetic factors during the molecular adsorption, migration, and coupling^89^. More recently, Wang et al. constructed NPG containing planar [18]annulene pores via hierarchical C–C coupling and cyclodehydrogenation by using the polyhalogen functionalized molecule with flexible skeletons as the precursor (Fig. 2f–h)^24^. Taken together, although in the above works, NPGs contained [18]annulene pores were successfully fabricated, it should be noted that the realization of highly ordered 2D networks is still very challenging. The factors that affect the quality of products can be summarized as follows: (1) the flexible building blocks prompt the similar strain energies of different conjugations, which influences the product selectivity; (2) the low-mobility of large precursors results in small-sized effective conjugation domains; (3) the polyhalogenated functional groups of precursors possess a wide dehalogenation temperature window, which induces the stepwise cleavage of C–X (X = Cl, Br, and I) and thus results in the products disordered; (4) the high reaction temperature results in non-selective dehydrogenation coupling, which leads to the formation of disordered by-products. In addition, the debromination step may also intersect with the cyclodehydrogenation step, which results in the debrominated sites quenched by hydrogen atoms, leading to the non-selective bond connection.

**Fig. 3:**
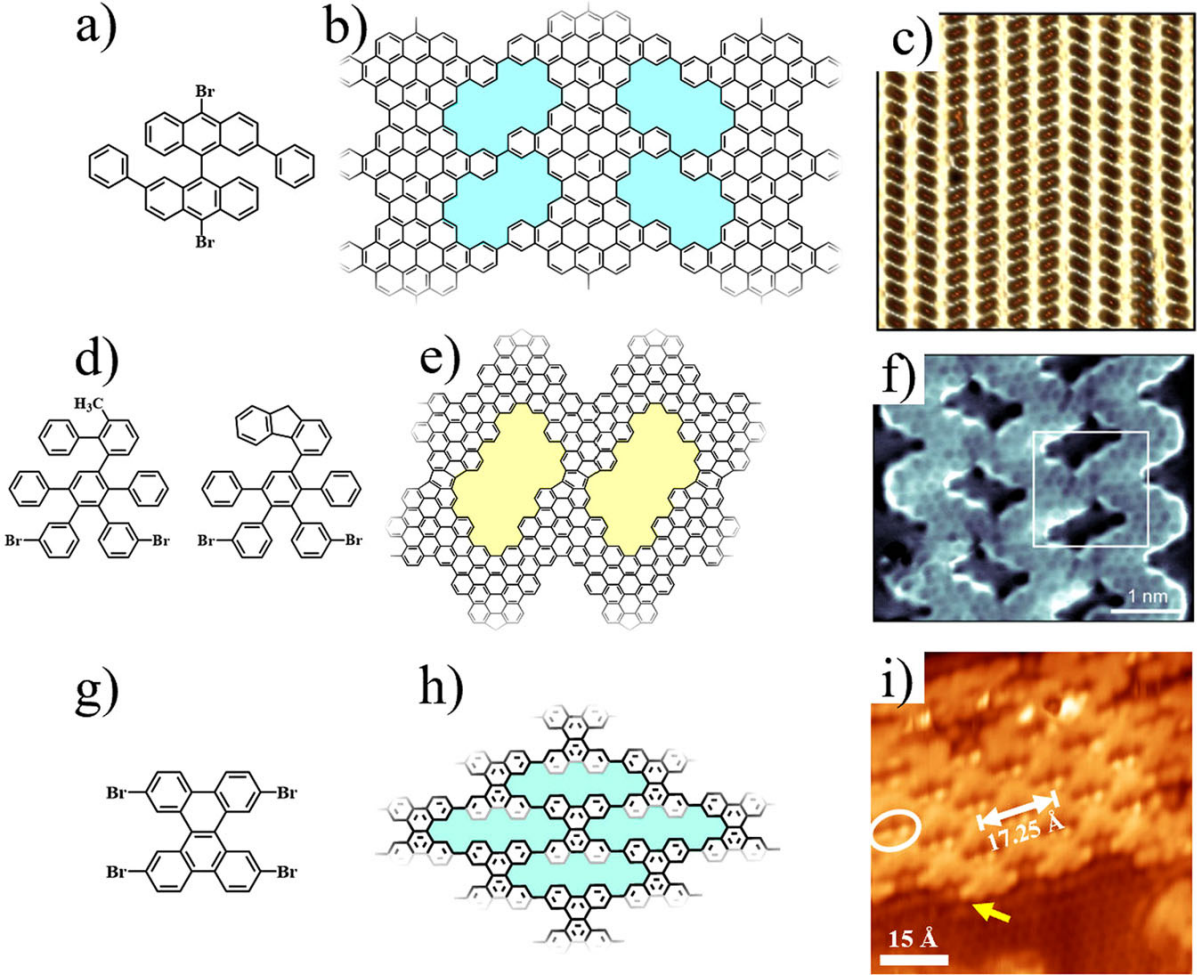
Structural models and STM images of some typical NPGs formed via the lateral fusion of GNRs or polymers. **a**–**c** On-surface synthesis of Moreno’s NPG, whose molecular structure is shown in (**b**) via three thermal-activated hierarchical reactions by using diphenyl–10,10′ dibromo-9,9′-bianthracene precursor (**a**) on Au(111), as revealed by the STM image in (**c**). The size of **c** is 18 × 18 nm^2^. (adapted with permission from ref. ^81^ ©2018 The American Association for the Advancement of Science). **d**–**f** On-surface synthesis of C-NPG whose molecular structure is shown in (**e**) via two-step hierarchical reactions by using two precursors (**d**) on Au(111), as revealed by the STM image in (**f**). (adapted with permission from ref. ^51^ ©2020 American Chemical Society). **g**–**i** On-surface synthesis of porous [30]annulene graphene nanosheet whose molecular structure is shown in (**h**) via Ullmann coupling and lateral fusion by using 2,7,10,15tetrabromodibenzo[a,c]triphenylene precursor (**g**) on Au(111), as revealed by the STM image in (**i**). (adapted with permission from ref. ^93^ ©2023 John Wiley & Son).

To date, in addition to the direct construction of [18]annulene porous NPG from small building blocks, the lateral fusion of GNRs on surfaces has also been utilized to fabricate NPG^81,83,92,93^. The surface-assisted synthesis by NPG from Moreno et al. represents a significant milestone (Fig. 3a–c)^81^. They achieved for the first time the synthesis of high-quality, large-size NPG (referred to as Moreno’s NPG) on Au(111) by exquisitely designing the rational precursor and precisely controlling the experimental parameters. Their synthesis strategy involved three thermal-activated hierarchical reaction steps: polymerization for constructing 1D chains through Ullmann coupling, aromatization for fabricating GNRs via cyclodehydrogenation reaction, and the formation of NPG through dehydrogenation cross-coupling. More recently, they introduced phenylene bridges to substitute the C–C bridges in the aforementioned NPG, which act as the chemical knobs through the continuous conformational transformation to modulate the interribbon coupling strength^83^. Jacobse et al. creatively constructed a new fully conjugated NPG using similar two-step hierarchical reactions^51^. The process involved the formation of initial GNRs, followed by laterally fusing benzene rings through five-membered rings to fabricate the rubicenetype bridging 2D chevron-type NPG (C-NPG) (Fig. 3d–f). The fully conjugated NPG induces a new low-energy state that is localized on the rubicene-type interfaces. In comparison to the NPG containing [18]annulene pores, there has been a significant improvement in the quality and size of Moreno’s NPG and C-NPG, which is essential to their applications in electronic devices^17^.

Very recently, our group reported the selective synthesis of NPG nanosheet containing periodic nonplanar [30]annulene pores on Au(111) (Fig. 3g–i)^93^. The planarity/non-planarity of [18]annulene pore and [30]annulene pore is determined by their width. [30]Annulene pore typically displays a nonplanar character owing to the huge steric hindrance between adjacent hydrogen atoms, whereas [18]annulene pore exhibits a planar character attributed to the larger pore width. The selective construction of nonplanar pores relies on the kinetics-driven debromination process, which is the rate-limiting step in the initial C–C coupling reaction.

The key to achieving a high-quality and ordered GNRs-fused NPG lies in two aspects: (1) The ingeniously designed precursors, with suitable mobility and debrominated sites, enable the initial polymerization of long-range ordered intermediate chains. Importantly, the steric hindrance promotes the high-selective activation of specific C–H bonds of intermediate GNRs during the dehydrogenative coupling step, allowing for selective lateral fusion. (2) An appropriate sample preparation strategy is crucial. The quality of 2D NPG was significantly improved when the precursors were deposited on the preheated substrate compared to that obtained by depositing the precursors on the RT substrate followed by stepwise annealing. The differences in product quality in different preparation strategies can be attributed to the full debromination induced by high temperature and the low molecule density during the coupling step, allowing for the length extension of intermediate polymers and subsequent lateral fusion to form the products with high selectivity and yields.

**Fig. 4:**
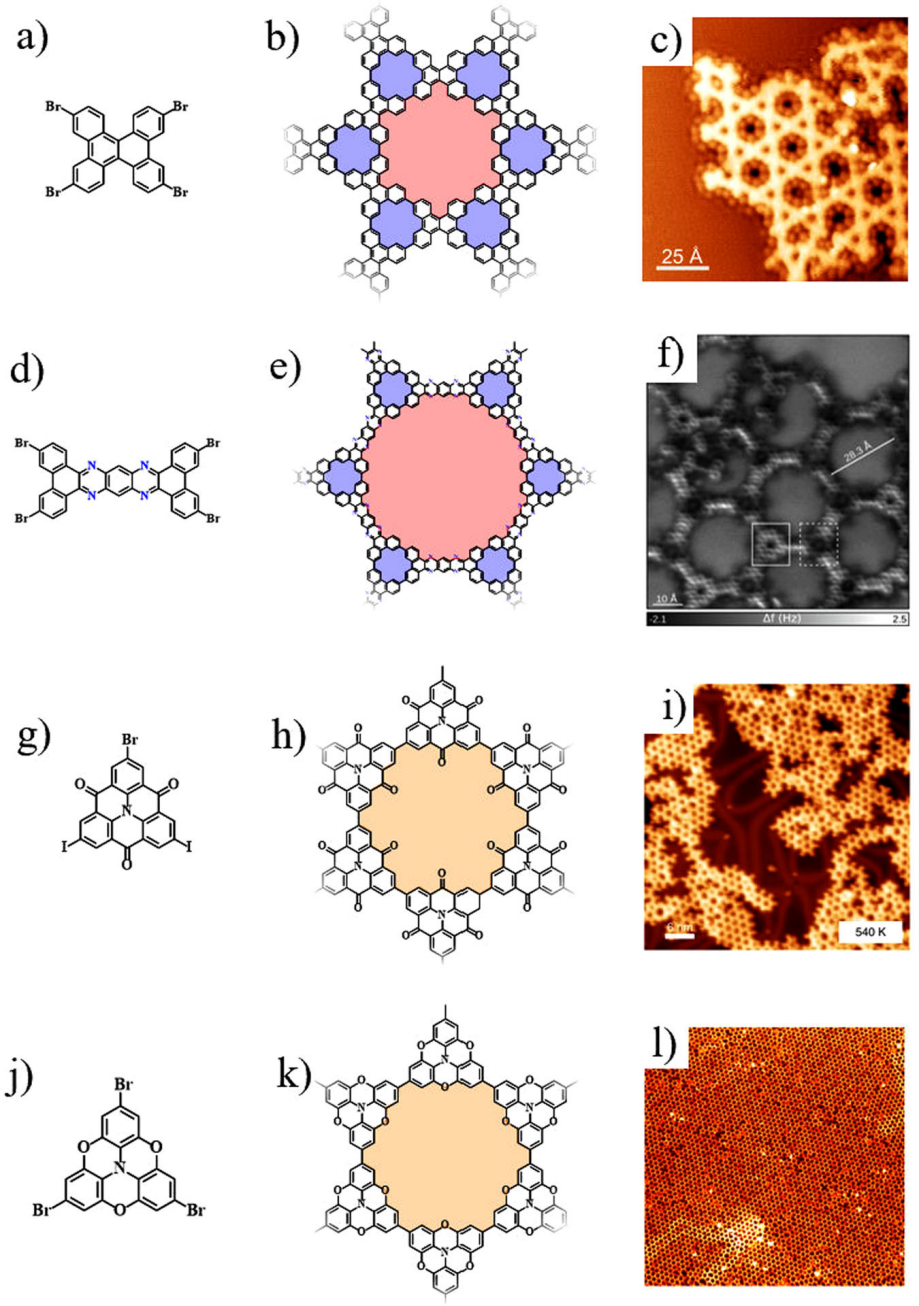
Structural models and STM images of some Kagome NPGs. **a**–**c** On-surface synthesis of Kagome nanoporous graphene whose molecular structure is shown in (**b**) via surface-assisted Ullmann coupling by using TBDTP precursor (**a**) on Ag(111), as revealed by the STM image in (**c**). (adapted with permission from ref. ^102^. ©2022 The Royal Society of Chemistry). **d**–**f** On-surface synthesis of nitrogen-doped Kagome nanoporous graphene whose molecular structure shown in (**e**) via surface-assisted Ullmann coupling by using 3,6,14,17-tetrabromodibenzo[a,c]dibenzo[5,6:7,8]quinoxalino-[2,3-i]-phenazine precursor (**d**) on Ag(111), as revealed by the STM image in (**f**). (adapted with permission from ref. ^103^. ©2021, with permission from John Wiley & Son). **g**–**i** On-surface synthesis of HTs-based Kagome NPG whose molecular structure is shown in (**h**) via surface-assisted Ullmann coupling by using carbonyl-bridged triphenylamines precursor (**g**) on Au(111), as revealed by the STM image in (**i**). (adapted with permission from ref. ^104^. ©2017 Springer Nature). **j**–**l** On-surface synthesis of HTs-based Kagome NPG whose molecular structure is shown in (**k**) via surface-assisted Ullmann coupling by using tribromotrioxaazatriangulene precursor (**j**) on Au(111), as revealed by the STM image in (**l**). The size of **l** is 90 × 90 nm^2^. (adapted with permission from ref. ^105^. ©2020 Springer Nature).

Apart from the NPG containing periodical nanopores of equal size, Kagome NPG, bearing regularly distributed triangular and hexagonal pores, has attracted increasing interest due to its fascinating electronic properties, especially flat band induced by the topologic frustration^94^. The Coulomb interaction becomes critical because the kinetic energy of the electron is quenched in the flat band and further induces the ferromagnetism, superconductivity, and quantum hall effects^95–97^. To date, the surface-assisted synthesis of Kagome lattice has primarily focused on systems stabilized by weak interactions (such as Van der Waals interaction, hydron bond or π–π stacking) and/organometallic coordination interactions^98–101^. Synthesizing Kagome NPG connected by fully covalent bonds continues to be an enormous challenge. Recently, we reported the successful synthesis of a pristine Kagome nanoporous graphene via Ullmann coupling, with the elaborately designing of the precursor 2,7,10,15-tetrabromodibenzo [g,p]-chrysene (including molecular symmetry, size, building block, and halogen substituent sites) (Fig. 4a–c)^102^. We demonstrated that the organometallic intermediate template played a critical role in the formation of covalent Kagome nanoporous NPG. A series of thermodynamic and kinetic strategies were used to optimize the quality and yield of Kagome NPG. The formation of a rhombic network and Kagome lattice are the two competing reaction pathways. The thermodynamically favored organometallic Kagome intermediate could be selectively fabricated by the deposition of precursors on a preheated substrate with a low deposition rate. Pawlak et al. fabricated a nitrogen-doped Kagome nanoporous graphene via the hierarchical Ullmann polymerization (Fig. 4d–f)^103^. Combined with density functional theory (DFT) calculations and scanning tunneling spectroscopy (STS), they found that the semiconducting character of nitrogen-doped Kagome graphene was induced by nitrogen doping, and the flat bands emerged near the Fermi level. In order to overcome the steric hindrance of near H atoms, the trigonal nodes twisted about 20–30°. The strain-induced non-planar character might limit the N-doped Kagome to form a large periodic network.

The honeycomb-Kagome lattice with heterotriangulenes (HTs) nodes was theoretically expected to possess a Dirac cone sandwiched between two flat bands^84^. It was suggested that when the center site of HTs in 2D Kagome NPG was embedded by an N atom, one flat band near the Fermi level remained flat and did not contribute to charge transport, while the other one exhibited strongly dispersed and supported highly mobile charge carriers. Functional groups (such as CO, O, or CH_2_) were employed to stabilize the HTs and achieve a cationic closed-shell conformation. Steiner et al. synthesized a 2D Kagome NPG through a hierarchical two-step synthesis, using HT with a N central atom and carbonyl bridge group as the node (Fig. 4g–i)^104^. Galeotti et al. constructed the high-quality and low-defect π-conjugated Kagome NPG on Au(111), whose nodes were occupied by two kinds of HTs blocks (with the oxygen and carbonyl bridge group) (Fig. 4j–l)^105^. Combined with angle-resolved photoemission spectroscopy (ARPES) and DFT calculations, they demonstrated the coexistence of Dirac cones and flat bands in the Kagome NPG containing the nodes with N central atom and oxygen bridge group. Notedly, the improvement of quality and order of Kagome NPG depends on the suitable precursor (rigid, highly symmetrical, and achiral) and proper sample preparation parameters (fine-tuned preheated deposition temperature and low deposition rate, promoting efficient surface diffusion and migration).

### Heteroatom-doped NPGs

Low-dimensional graphene nanostructures can be functionalized by chemical, physical, or combined methods to tune their properties and further satisfy the requirements of their various applications^106^. Aimed at the application of transistor devices and gas/ions separation membranes, large amounts of tuning strategies have been applied to functionalize the NPGs. Heteroatom doping represents one of the most feasible ways to functionalize NPG, as evidenced by the abundant examples in recent literature about functional porous graphene. Doping plays a critical role in tuning the electronic properties of NPGs, to enable band engineering by controlling the type and concentration of charge carriers^12^.

Nitrogen (N) and boron (B) are typical electron donors and acceptors in semi-conductors, respectively. Due to their similar atomic radius to carbon, N or B doping could readily fit into the lattices of NPG^9,107^. Moreover, the covalent bonds formed between N (and or B) and C atoms provide doped NPG with long-term thermal and chemical stability.

**Fig. 5:**
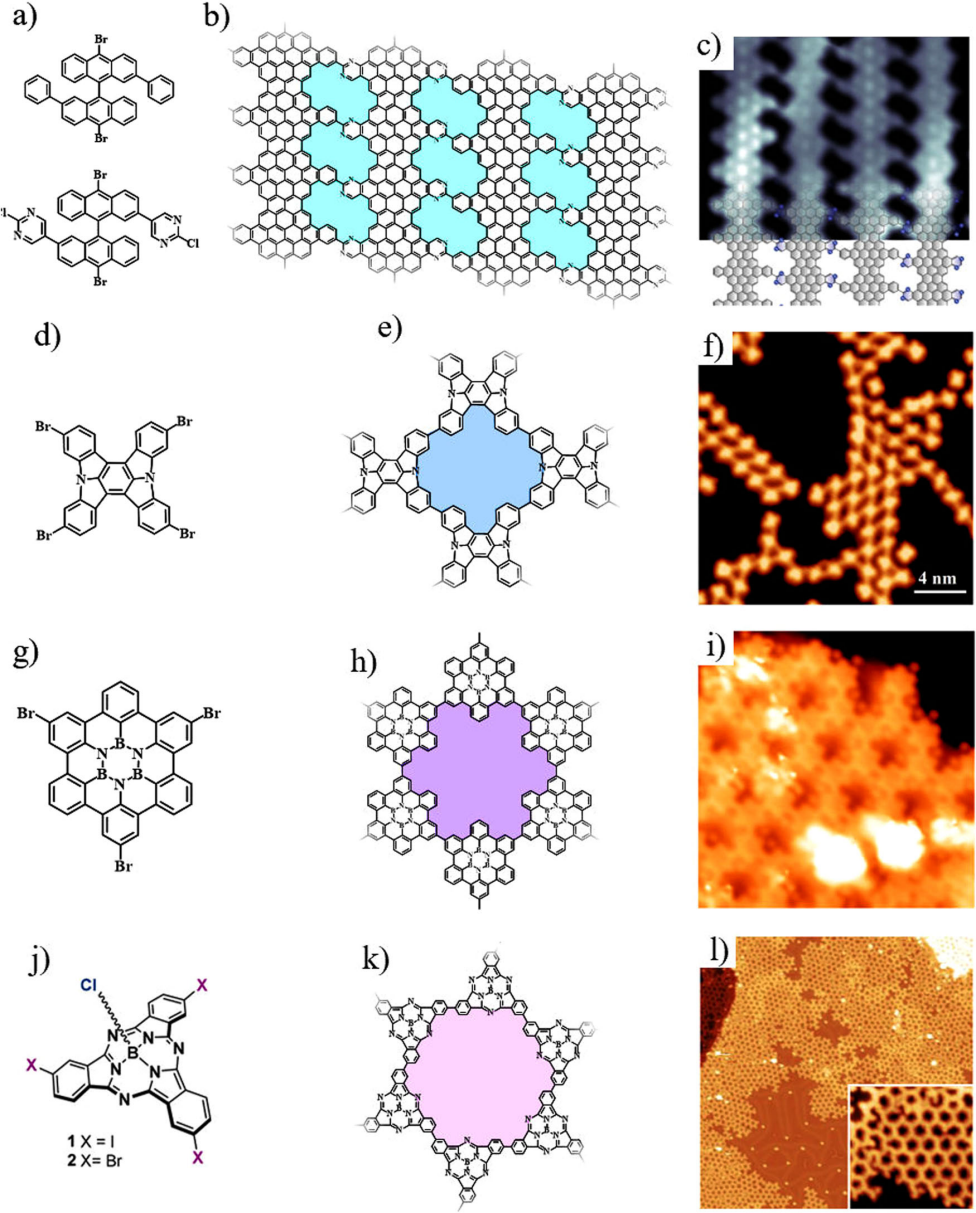
Structural models and STM images of some typical heteroatom-doped NPGs. **a**–**c** On-surface synthesis of NPG-based heterojunctions whose molecular structure is shown in (**b**) via the templet strategy and hierarchical reactions from two precursors (**a**) on Au(111), as revealed by the STM image in (**c**). The size of **c** is 7 × 6 nm^2^. (adapted with permission from ref. ^113^. ©2022 John Wiley & Son). **d**–**f** On-surface synthesis of nitrogen-doped NPG whose molecular structure is shown in (**e**) via surface-assisted Ullmann coupling by using DipICz precursor (**d**) on Ag(111), as revealed by the STM image in (**f**). (adapted with permission from ref. ^114^. ©2022 American Institute of Physics). **g**–**i** On-surface synthesis of NB-doped NPG whose molecular structure is shown in (**h**) via surface-assisted Ullmann coupling by using NB-doped precursor (**g**) on Au(111), as revealed by the STM image in (**i**). The size of (i) is 90 × 90 nm^2^. (adapted with permission from ref. ^123^. ©2015 American Chemical Society). **j**–**l** On-surface synthesis of NB-doped NPG whose molecular structure is shown in (**k**) via surface-assisted Ullmann coupling by using subphthalocyanines-based precursor (**j**) on Au(111), as revealed by the STM image in (**l**). The size of (**l**) is 100 × 100 nm^2^ (inset: 15 × 15 nm^2^). (adapted with permission from ref. ^124^. ©2022 American Chemical Society).

There are seven types of possible N doping in NPG: triazine, pyrazine, pyridine, phenanthridine, pyrimidine, carbazole, and graphitic N atoms^108^. N-doped NPG obtained via on-surface synthesis was first reported by Grill et al. in 2007, wherein porphyrin-based 2D networks were obtained via Ullmann coupling between the tetra(4-bromophenyl)porphyrin precursor molecules^109^. Shi et al. synthesized the NPG containing periodic triazine rings^110^. Several steps of hierarchical reactions, including alkynyl cyclotrimerization, C–O bond cleavage, and C–H bond activation, were used to fabricate the 2D network. Introducing pyrazine N into the building block during precursor design can induce electronic band offset at the C–N heterojunction and lead to the redistribution of local charge density^111,112^. The length of bridges, the concentration of pyrazine N atoms, and the framework structures can be adjusted to tune the band gaps. Based on the templet of initial GNRs, Tenorio et al. devised a novel template method and implemented hierarchical reactions to create lateral superlattice heterojunctions containing periodic nanopores and pyrimidine N atoms (Fig. 5a–c)^113^. Recently, by utilizing a precursor molecule containing pyrimidine N atoms, they explored the stability of N-heteroatoms in NPG and successfully synthesized NPG with pyrimidine-bridged graphene nanoribbons as the building block^108^. Moreover, an elaborately designed precursor called 3,7,12,16-tetrabromodibenzo[2,3:5,6]pyrrolizino[1,7-bc]indolo[1,2,3-lm]carbazole (DipICz), which contains graphitic N atoms, was used to directly introduce graphitic C–N into NPG (Fig. 5d–f)^114^. It is noteworthy that the molecular structure of the DipICz precursor is similar to that of TBDTP. The reason why the DipICz precursor doesn’t tend to construct Kagome nanoporous graphene may be the introduction of N-doped pentagonal rings, resulting in the deviation of the angle of C–C covalent sites from the requirements of an ideal Kagome lattice.

B is a typical electro-deficiency element and is widely used as a dopant in graphene^9,115–117^. Zwaneveld et al. first introduced B/O dopants into NPG to fabricate boroxine-linked and dioxaborole-linked covalent organic frameworks (COFs) via surface-assisted dehydration reactions, indicating the tunability of nanoporous sizes^118^. Graphene and BN sheets have commensurate parameters, making it feasible to introduce B and N atoms as co-dopants into graphene nanostructures, as extensively demonstrated in theories and experiments^119–122^. Sánchez-Sánchez et al. obtained the NPG containing periodic B and N atoms via surface-assisted Ullmann coupling and subsequent cyclodehydrogenation (Fig. 5g, h)^123^. Labella et al. fabricated the BN-doped NPG by employing an enantiopure precursor containing subphthalocyanines (Fig. 5j–l)^124^. The yield and quality of NB-doped NPG were improved through the low evaporation rate and high deposition temperature.

It should be noted that 2D COFs synthesized on surfaces also fall under the category of 2D porous materials containing heteroatoms. Compared to NPGs, COFs typically contain various heteroatomic functional groups and involve weaker electron conjugation and hybridization. Representative examples encompass nitrogen-containing COFs synthesized via Schiff base reactions^125^, boron/oxygen-containing COFs derived from boric acid condensation reactions^118^, and recently reported COFs incorporating 1,4-disilabenzene (C_4_Si_2_) linkers^126^. 2D COFs have been extensively discussed in previous reviews^127^.

### Recent advances in the application of NPG

NPG has demonstrated excellent performance in terms of novel electronic and optical properties^6^. It has been shown that NPG holds great promise for applications in FETs and carbon nanocircuitry^76,79–82,113,128^.

**Fig. 6:**
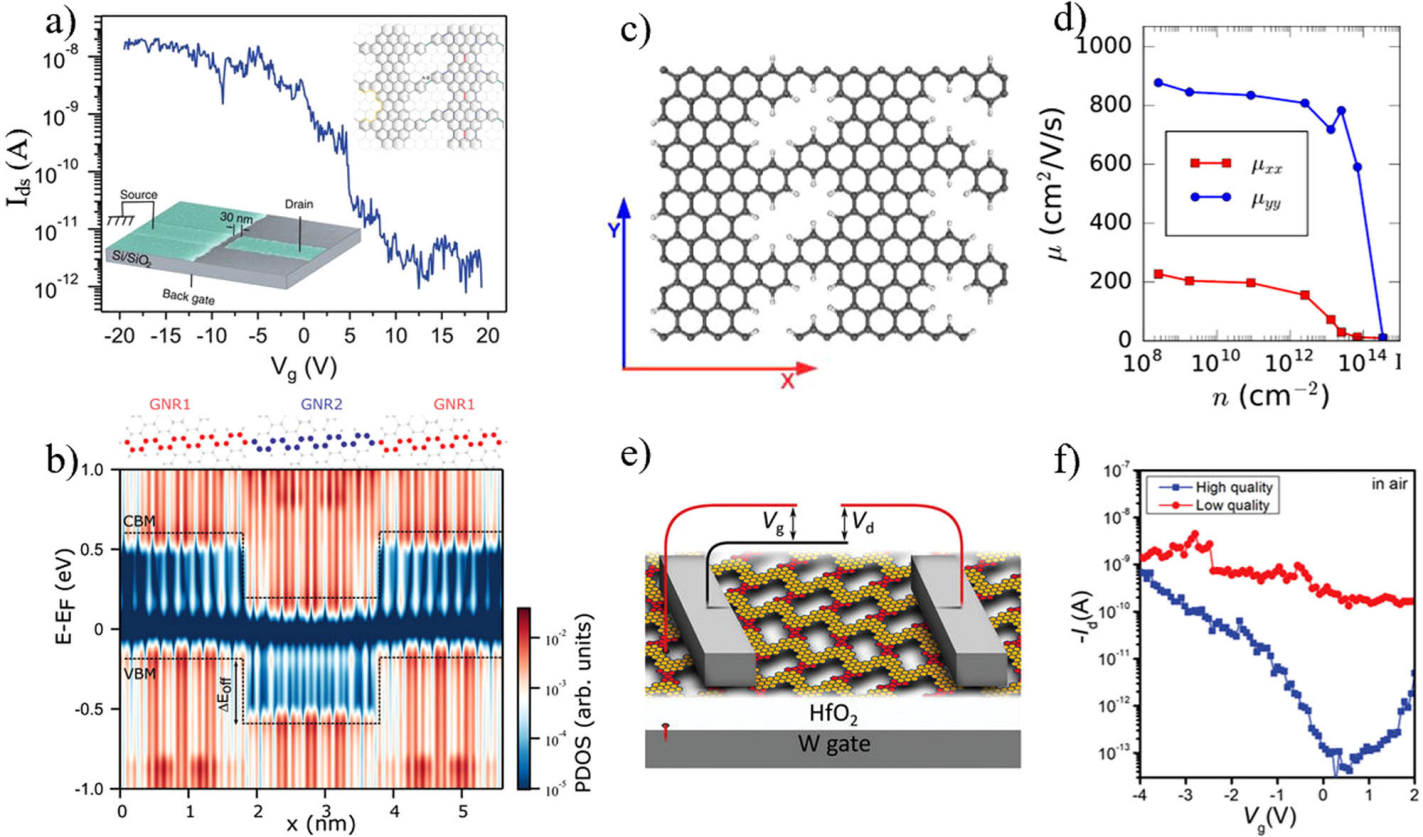
Electronic applications of NPGs. **a** *I*_d_–*V*_g_ characteristics of Moreno’s NPG device (30 nm channel length) gated by a SiO_2_ gate oxide (90 nm thick). (adapted with permission from ref. ^81^. ©2018 The American Association for the Advancement of Science). **b** Simulated LDOS scan across the heterojunction including GNR1-GNR2-GNR3 triplet. (adapted with permission from ref. ^113^. ©2022 John Wiley & Son). **c** The unit cell of the NPG model is marked by the *x*- and *y*-axis. **d** The intrinsic electron mobility along the two axes in (c), versus electron density. (adapted with permission from ref. ^79^. ©2019 IOP Publishing). **e** Schematically device structure of a local back-gated based on C-NPG FET. **f** Room temperature *I*_d_–*V*_g_ characteristics of the FET devices based on C-NPG in air. (adapted with permission from ref. ^128^. ©2021 John Wiley & Son.).

Previous theoretical and experimental studies demonstrated the effectiveness of GNRs applied in FETs^129–131^. According to previous work, NPG-based FETs exhibit higher *I*_ON_/*I*_OFF_ ratios compared to GNR-based FETs, which can achieve about 100 times stronger saturated currents than individual GNR-based FETs^6^. However, traditional top-down methods (such as lithography) and in-solution methods have reached a bottleneck in meeting the requirements for fabricating atomically precise NPG-based FETs. In recent years, elaborately designed reaction strategies have been suggested to achieve the atomically precise NPG relying on the on-surface synthesis^51,81,128^. Based on the advantages of large size and structural accuracy, Moreno’s NPG exhibited potential for FET application, showing the performance with hole transport and an *I*_ON_/*I*_OFF_ ratio of about 10^4^ (Fig. 6a)^81^. By introducing periodic heteroatomic atoms into Moreno’s NPG, chemically heterogeneous nanoporous junctions were obtained^113^. The coherent lateral superlattice heterojunctions induce the sharp band discontinuity, while the tunneling states exist on both sides of the junction (Fig. 6b). Moreover, coherent lateral superlattice heterojunctions with atomically sharp band discontinuity obtained via surface-assisted hierarchical coupling exhibited promising applications to the future optoelectronics and ion/gas sieving. Based on the system of Moreno’s NPG, several theoretical studies investigated the electronic properties and potential applications of this material^76,80,82^. Moreno’s NPG showed a strong anisotropy in carrier mobility, as 800 cm^2^ (VS)^−1^ at low carrier density (Fig. 6c, d)^79^. Lee et al. presented an inverse design of NPG/Graphene bilayers with electric-field-tunable bandgaps via breaking inversion symmetry or moving and merging Dirac points^80^.

The recent successful development of fully conjugated C-NPG has sparked new interest in FET research (Fig. 6e)^51,128^. The interface electronic states, localized on the rubicene-type bridge connecting the single-strand nanoribbons, contribute to the low-energy band and make this fully conjugated C-NPG well-suited for FET applications. The *I*_ON_/*I*_OFF_ ratios of the C-NPG FETs exceed 10^4^ (Fig. 6f). Thus, the atomically precise structures and tunable pores of NPG, fabricated via on-surface synthesis, offer potential for FET applications.

The presence of nanopores in NPG leads to local confinement of π-electrons, enabling GNRs to serve as 1D electronic nanochannels that allow preferential electron movement. For the system of Moreno’s NPG, Calogero et al. theoretically predicted the interference Talbot effect of electrons’ propagation in NPG and experimentally explored the Talbot effect by dual-probe scanning tunneling microscopy^76^. Because the C–C bonds bridging the GNRs in Moreno’s NPG facilitate the rapid spatial dispersion of electronic currents, other cross-linking modes to bridge the GNRs in NPG were explored, i.e., benzene bridges with either para or meta connections, resulting in confined current within GNRs with a width of 0.7 nm^82^. Through rational framework design, crosstalk between GNRs in NPG is effectively blocked, allowing for electronic isolation.

Both theoretical and experimental studies of integrating NPG into electronic devices have been explored. The realization of these practical applications requires the transfer of low-dimensional graphene-based materials from metallic substrates to insulating substrates (such as SiO_2_ and Al_2_O_3_). Two relatively mature strategies have been employed in previous works for the transfer of GNRs: (1) wet-etch methods, wherein GNRs are initially grown on an insulating substrate covered with a single crystal Au film, followed by Au etching and transfer to the insulating substrates^132^; (2) electrochemical delamination, by direct growth of GNRs on Au single crystal substrates, with poly(methyl methacry-late) (PPMA) as the support layer, and transfer through electrochemical delamination^133^. Modifications and optimizations to these methods have been developed to maintain structural integrity during the transfer of GNRs to insulating substrates^129^. Recently, the wet-etch methods were used to transfer C-NPG from gold/mica substrates onto prepatterned device substrates^128^ (Fig. 6e). The successful fabrication of FETs with C-NPG has suggested its advantages over GNRs, i.e., high-yield transfer onto dielectric surfaces, and realizing uniformity without suffering from lateral displacement and aggregation effects.

Fabrication of NPGs directly onto insulating substrates facilitates NPG’s practical electronic applications by avoiding the annihilation of their semi-conductive and spin properties due to a strong interaction between NPG and metal substrates. However, the weak adsorption of molecules on nonmetallic surfaces typically results in heavy molecular desorption during the annealing process. Photo-driven strategies on insulating substrates provide an approach to overcome this limitation. It has been demonstrated that photo-driven strategies can trigger the activation of active groups on surfaces and induce some surface reactions at a mild temperature^134,135^. In specific organic reactions, photo-driven strategies can avoid the side reactions caused by heating, presenting a promising strategy for the fabrication of low-dimensional carbon-based materials.

### Conclusion and outlook

Both theoretical and experimental investigations have demonstrated the advantages of NPGs in various nanotechnology applications. With its unique combination of tunable nanopores and single-atom thickness, NPGs exhibit potential in electronic applications such as FETs, carbon nanocircuitry and so on. The tunability of nanopore size, shape, edge geometry, and periodicity allow for the customization of the NPGs’ bandgap, making NPGs particularly well-suited for FETs. Moreover, high electron mobilities of NPGs induced by specific nanopore configurations enable the rational design of carbon nanocircuitry. The above-mentioned applications are all sensitive to the geometric structures of NPGs, which require atomically precise and low-defect NPG fabrication.

On-surface synthesis, as one of the most effective methods for fabricating atomically precise and highly ordered NPGs, has attracted intensive attention. Combined with powerful surface analysis techniques, such as STM/STS, XPS, and ARPES, the morphology, electronic properties, bond structures, and synthetic mechanisms of NPGs can be explored in-depth. In the field of on-surface synthesis of NPG, the [18]annulene pores were first introduced into NPG. Later on, Moreno’s NPG and the fully π-conjugated C-NPG were synthesized via the lateral fusion of atomically precise GNRs. The geometric structure of NPG plays a critical role in determining its properties. Theoretical predictions have suggested the existence of Dirac cone and flat bands in Kagome NPG structures. A few studies constructed NPG with Kagome lattices on surfaces and explored their electronic properties. Functionalizing NPGs through heteroatomic doping offers a way to modify the building blocks of the nanopores and tune the physical properties. N- or/and B-doped NPGs, synthesized through surface-assisted methods, have emerged as a new platform for exploring 2D lateral heterojunctions and investigating the electronic and photonic properties of NPG.

Increasing size and reducing defects through effective synthetic methods are crucial to facilitate the applications of NPGs. At present, most of the NPGs are fabricated through surface-assisted Ullmann coupling reactions. Designing the precursors with two halogen isosubstituents requires rational design of building blocks since achieving the highly selective reaction at high temperature needs to trigger the lateral fusion of GNRs at the equivalent reaction sites, thereby avoid non-selective bonding^81^. Starting from precursors with three halogen isosubstituents, the formation of NPG only involves the C–C coupling after dehalogenation. At this stage, symmetry and rigidity play crucial roles in the formation of high-quality networks^105^. The use of more flexible building blocks leads to a similar reaction energy barrier during the formation of irregular pentagons, hexagons, and heptagons, which in turn induce defects. In the case of precursors with four halogen substituents, it is important to consider increasing the selectivity of the required reaction pathway through the employment of proper thermodynamic and kinetic strategies. This is necessary because there are multiple possible pathways that can arise from the multiple radical sites of dehalogenated molecules^102^.

NPGs with various geometric structures have been theoretically predicted, but few of them have been implemented in experiments, which limits the exploration of new physical properties. Here are three prospectively surface-assisted strategies for producing NPG with novel properties in the future. Firstly, exploration of more accessible on-surface reactions beyond Ullmann coupling could lead to the construction of NPG with new topological structures. Secondly, expanding methods to introduce periodic nonplanar pores in NPG. For example, the twisted polycyclic aromatic hydrocarbons exhibit quite different electronic band structures compared to their planar counterparts. Thirdly, the introduction of nonhexagonal rings requires further investigation, as it induces local strain and electron redistribution within the building blocks. These structures may exhibit exotic electronic, magnetic, and quantum-coherent properties. Apart from expanding the category of NPG with novel physical properties, foundational works for optimizing the quality of NPG along with more efficient transfer methods are needed to be done before their commercial applications. In particular, the large-scale fabrication of NPG requires the understanding of reaction mechanisms at the single-molecule level. The interpretation on the relationship between NPG structure and electronic properties asks for the efforts of theoretical chemists and physicists. In addition, the development of efficient transfer methods needs collaboration between scientists in the fields of surface science and nanofabrication.

Finally, the metal surface may severely quench the intrinsic electronic properties of low-dimensional carbon nanostructures. Thus, for NPG materials to be applicable in real electronics, a critical step is to transfer the formed NPG from the metal crystal onto insulating substrates to create NPG-based devices. The main challenges during the transfer process of NPGs are to avoid undesired contamination and/or deterioration of the NPGs and to reduce costs. Fabrication of NPG directly onto nonmetal substrates offers a promising solution to circumvent the undesired contamination and the damage of structure in transfer processes. According to previous works, H–F zipping^136^, cyclodehydrogenation, and Ullmann coupling reactions^70^ have been triggered on nonmetallic substrates^137^. This method also provides a new approach for the fabrication of NPG and integrating electronic devices on nonmetallic substrates. Addressing the issues of weak molecular adsorption and low reaction selectivity is the key to the fabrication of NPGs on nonmetallic surfaces. A potential option to enhance the adsorption of molecules on nonmetallic surfaces is the introduction of co-adsorbates or adsorptive group^138^, such as carboxylic acid^139^. A deeper understanding of the reaction mechanism can help to enhance reaction selectivity by employing rational thermodynamic or kinetic strategies. Photo-driven reactions are also a potential strategy for the synthesis of NPGs on nonmetallic substrates, by which heating-induced desorption and side reactions could be avoided. It is believed that with the increasingly developing and expanding research interests in NPG, an in-depth understanding of their fundamental properties and extensive exploration of their synthetic strategies will further promote their applications in future nanoscience and nanotechnology.
